# Image routing via atomic spin coherence

**DOI:** 10.1038/srep18179

**Published:** 2015-12-10

**Authors:** Lei Wang, Jia-Xiang Sun, Meng-Xi Luo, Yuan-Hang Sun, Xiao-Xiao Wang, Yi Chen, Zhi-Hui Kang, Hai-Hua Wang, Jin-Hui Wu, Jin-Yue Gao

**Affiliations:** 1 Key Laboratory of Coherence Light, Atomic and Molecular Spectroscopy, College of Physics, Jilin University, Changchun, 130012, P. R. China; 2 Institute of atomic and Molecular Physics, Jilin University, Changchun, 130012, P. R. China

## Abstract

Coherent storage of optical image in a coherently-driven medium is a promising method with possible applications in many fields. In this work, we experimentally report a controllable spatial-frequency routing of image via atomic spin coherence in a solid-state medium driven by electromagnetically induced transparency (EIT). Under the EIT-based light-storage regime, a transverse spatial image carried by the probe field is stored into atomic spin coherence. By manipulating the frequency and spatial propagation direction of the read control field, the stored image is transferred into a new spatial-frequency channel. When two read control fields are used to retrieve the stored information, the image information is converted into a superposition of two spatial-frequency modes. Through this technique, the image is manipulated coherently and all-optically in a controlled fashion.

A future quantum information network would consist of some separated devices, in which quantum information can be stored and manipulated in a controlled fashion. Coherent interactions between light and matter provide powerful tools to obtain the above goal. As a prominent example, electromagnetically induced transparency (EIT) has been successfully applied to manipulate the quantum states of the light fields^1^. The unique properties of EIT allow for a wide range of coherent light-matter phenomena^2^. In particular, an EIT system can be used as an important candidate for storing light pulse^3^,^4^. In the EIT system, the propagation of light fields can be described by coupled light-matter excitations termed as dark-state polariton^5^. By manipulating the Rabi frequency of the control field, the probe light can be converted into atomic spin coherence, and vice visa. Furthermore, this EIT-based light storage is proved to satisfy quantum storage requirements, and the quantum properties of the light fields can be preserved during the storage operation^6^,^7^,^8^,^9^.

Coherent and all-optical manipulation of image plays a significant role in many fields, including holography, classic and quantum correlations, image and information processing^10^. Most experiments of EIT-based light-storage deal with the amplitude and phase variation of the probe pulse in the time domain, and pay little attention to transverse spatial image carried by the probe pulse. Further applications require the storage of two-dimension image with large information intensity. Recently, some processes related to image storages based on EIT effect have been experimentally performed^11^,^12^,^13^,^14^,^15^,^16^,^17^,^18^,^19^,^20^. The slowing and storage of image has been obtained in EIT-driven experimental mediums^11^,^12^,^13^. The storage and retrieval of the image carried by thermal light have been reported in atomic gases^14^,^15^. The storage of light beams carrying orbital angular momentum has been demonstrated experimentally^16^,^17^,^18^. Frequency conversion of stored image has been performed in cold atoms^19^. The preservation of transverse spatial coherence in image storage has been discussed by the spatial interference fringes^20^.

The coherent manipulation of the image in EIT-driven mediums is very useful for information processing. Transfer and splitting of the image will enrich the manipulation technique of the image, and be important for further image processing. In this work, we experimentally demonstrate a controllable spatial-frequency routing of image via stored atomic spin coherence in an EIT-driven solid. Compared with atomic gases, solid-state mediums have more practical applications in information processing. This image routing is based on EIT-based light storage. Under the EIT condition, a transverse spatial image carried by the probe pulse is mapped into atomic spin coherence by switching off the write control field, and later retrieved through the opposite operation. By manipulating the frequency and spatial propagation direction of the read control field, the stored image is transferred into a new spatial-frequency channel. When two read control fields are applied to read out the stored information, the image information is distributed simultaneously into its initial and transferred information channels. The intensity and similarity of the transferred image compared with that of the initial channel are further analyzed. Such spatial-frequency image routing can be used for routing image information and linking different information channels and devices, and has important applications in the fields of image processing and information network.

## Results

**Figure 1:**
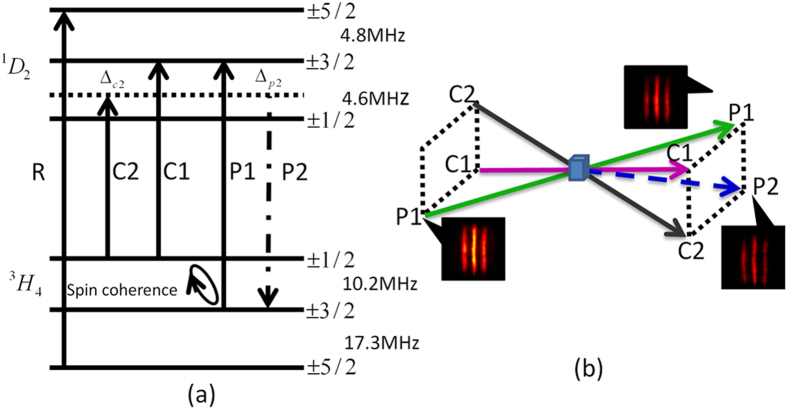
(**a**) The coupling scheme of Pr ions. (**b**) The experimental schematic diagram of the image routing. The powers of the control-1, control-2, probe-1 and repump field are 10 mW, 9 mW, 0.6 mW and 5 mW, respectively.

The image storage and routing are performed by using Pr^3+^: Y_2_SiO_5_ (Pr: YSO) crystal, as depicted in Fig. 1(a). Rare-earth-doped solids without atomic diffusion provide spatially-fixed interaction units^21^,^22^, and can be an excellent candidate for information processing^23^,^24^. The control-1 field 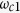 is resonant with the 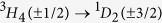
transition, and the probe-1 field 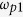 is resonant with the 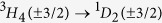
transition. These two fields prepare atomic spin coherence between two lower levels by light storage. An additional control-2 field 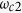 is used to scatter stored atomic spin coherence to generate the probe-2 field 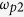. The newly-generated probe-2 field has a new frequency as well as a new spatial direction, and spatial-frequency routing is realized. The repump field 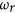 couples the 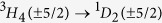 transition, and is used to pump the populations to the levels 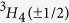 and 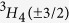. The optical inhomogeneous broadening is several GHz, and the spin inhomogeneous broadening of 10.2 MHz is about 30 KHz. The experimental schematic diagram is shown in Fig. 1(b). The angle between laser beams is about 85 mrad. The dopant concentration of Pr:YSO crystal is 0.05%.

**Figure 2:**
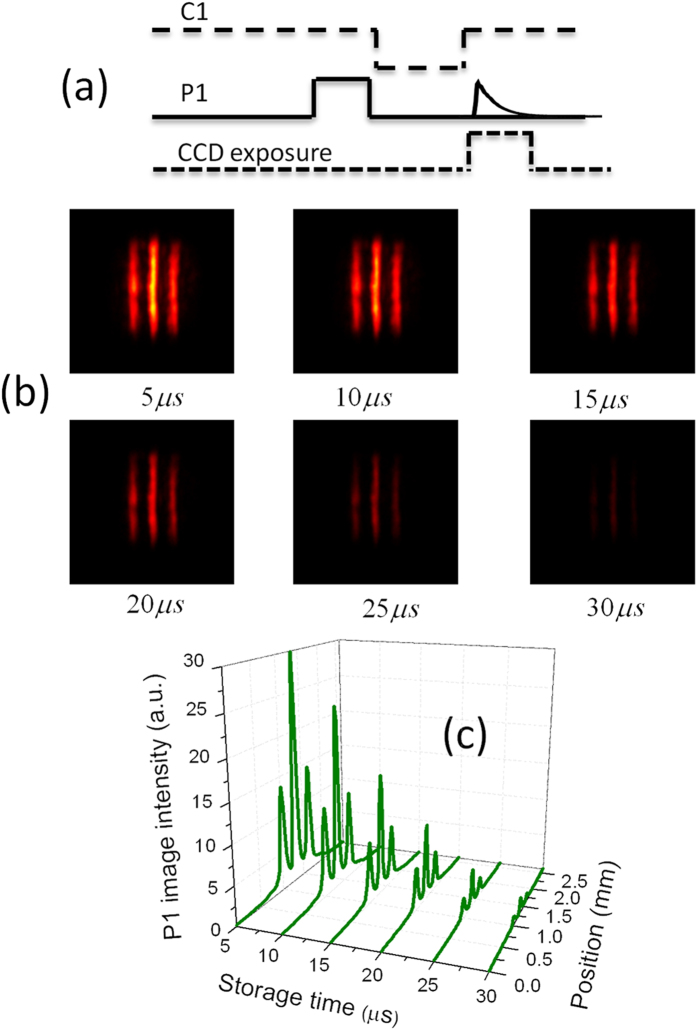
(**a**) The pulse sequences of light storage demonstration. (**b**) The retrieved probe-1 images for different storage times. (**c**) The intensity profile of the retrieved probe-1 image versus the storage time.

By using the preparation pulses as described in ref. 25, Pr ions shown in Fig. 1 are prepared, and the populations are pumped to 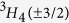 level. Subsequently, the 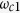 field coupling 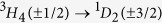 transition and the 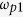 field coupling 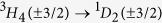 transition are applied to form an EIT lambda system. The pulse sequences of the storage demonstration are detailed in Fig. 2(a). By switching off the control-1 field, the probe-1 pulse is mapped into atomic spin coherence between two lower levels. After a certain storage time, by switching back on the control-1 field, stored atomic spin coherence is retrieved into the initial probe-1 pulse. Transverse image is expected to be stored and retrieved by this EIT-based storage. A transverse spatial image is generated by using a mask of three-stripe imprinted on the probe-1 pulse. The CCD camera is used to analyze the transverse spatial profile, and is triggered after the control-1 field is switched back on in the retrieval process. The exposure time of CCD is made to match the width of the retrieval probe-1 pulse to efficiently record the retrieved image. Figure 2(b) shows the storage and retrieval of three-stripe image for different storage times. It is seen that the retrieved images preserve the initial three-stripe image. To quantitatively discuss the retrieved image, the image intensity distribution in the horizontal direction is analyzed by image software. The retrieved image intensity profile versus the storage time is shown in Fig. 2(c). Three intensity peaks correspond to three stripes of the retrieved image. It is seen that the retrieved image intensity decreases with the storage time. The 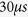 decay is due to the dephasing of atomic coherence induced by the inhomogeneous broadening of the spin transition. Dynamical decoupling techniques and specific magnetic field orientations^23^,^24^ can be used to increase the storage time. In image storage of atomic gases, atomic diffusion makes the retrieved image diffuse^11^. This diffusion effect does not occur in solids. Thus, in our case, the retrieved image does not exhibit the diffusion behavior with the storage time, only the retrieved image intensity decreases.

**Figure 3:**
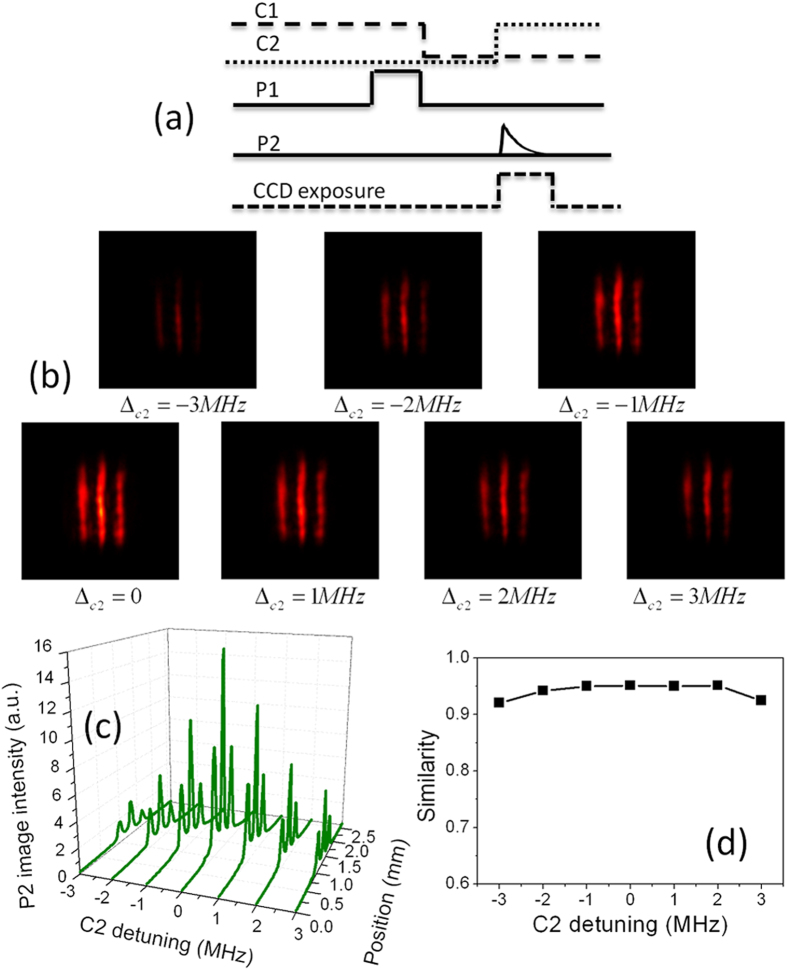
(**a**) The pulse sequences of retrieving probe-2 image. (**b**) The retrieved probe-2 images for different detunings of the control-2 field at 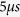 storage time. (**c**) The intensity profile of the probe-2 image versus the control-2 detuning. (**d**) The similarity between transferred probe-2 and probe-1 images versus the control-2 detuning.

In the above storage, the initial control-1 field is used to read out the stored information, and the retrieved optical image has the same carrying frequency and spatial propagation direction as the initial image. There have been some experiments dealing with the retrieval of the stored pulse in a new frequency channel^25^,^26^,^27^,^28^, where the probe pulse does not carry the spatial image. We focus that whether the stored transverse image can be retrieved in a new spatial-frequency channel. This image routing relies on the use of the new read control field. The pulse sequences of image routing are shown in Fig. 3(a). In the retrieval process, a new control-2 field instead of the control-1 field is used to read out the stored information. Due to the interaction between the control-2 field and atomic spin coherence, a new probe-2 pulse is obtained. The essence of this image routing is four-wave mixing based on stored atomic spin coherence. The generation of the probe-2 field satisfies two-photon resonance (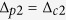) and phase-matching condition (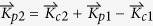), as shown in Fig. 1. Thus the frequency and the spatial propagation direction of the probe-2 image can be controlled by that of contro-2 field. Figure 3(b) shows the retrieved probe-2 images recorded by CCD camera for different detunings of the control-2 field. It is found that the transverse profile of the retrieved probe-2 pulse preserves the initial three-stripe image. This is because that the medium is coherently prepared in advance by light storage, the newly-generated probe-2 field will copy the transverse structure of the probe-1 field. It is noted that the retrieved probe-2 image not only has a new carrying frequency, but is also spatially separated from the input probe-1 image. By using cold atoms, Ref. 19 has showed that the stored image could be transferred into a new frequency mode. Here, by using a doped solid, we demonstrate that the retrieved image has a new spatial propagation mode as well as a new frequency mode.

The quality of the transferred probe-2 image is further analyzed. Figure 3(c) shows the intensity profile of the probe-2 image versus the detuning of the control-2 field. The retrieved image intensity is related to the detuning of the control-2 field, and the maximum intensity of the retrieved image corresponds to zero detuning of the control-2 field. From Fig. 3(b), it is seen that the transferred probe-2 image has a little distortion compared with the probe-1 image, which mainly comes from the imperfect overlap between the control-2 field and the interaction region. In order to check the quality of transferred probe-2 image, we further analyze the similarity R of the transferred probe-2 image compared with the probe-1 image. The similarity R is calculated by using the formula of Ref. 17, 
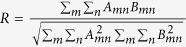
, where 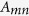 and 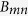 are the gray-scale intensities recorded for pixels m and n of the two images to be compared. Figure 3(d) shows the similarity between two images versus the detuning of the control-2 field. All obtained similarities are above 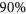, which indicates that the transferred image has the high quality compared with that in the initial channels. The similarity has weak dependence on the control-2 detuning, although the probe-2 image intensity is related to the control-2 detuning. The little variation of the similarity versus the control-2 detuning comes from the impact of the image background noise. The background noise is mainly from the scattering light of the experimental environment and vibration noise of experimental devices. Because two images have different spatial propagation directions, the background noises of two images are different. In the case of weak probe-2 image intensity, the transferred image has a low signal-noise ratio, and the impact of background noise is enhanced. If ignoring the background noise, the similarity is expected to keep constant. The similarity mainly depends on the overlap between the control-2 field and stored atomic spin coherence. By carefully adjusting the position of the control-2 field in the crystal, we obtain the good overlap. When we change the control-2 detuning, the position of the control-2 field in the crystal is almost kept unchanged. So the similarity is expected to remain constant when the control-2 detuning changes.

**Figure 4:**
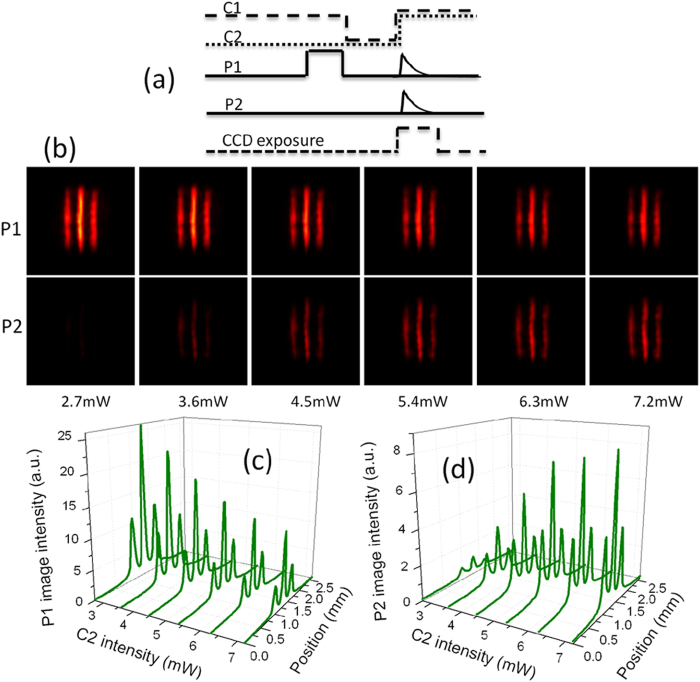
(**a**) The pulse sequences of simultaneously retrieving probe-1 and probe-2 images. (**b**) The probe-1 and probe-2 images for different control-2 intensities at 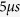 storage time. (**c**,**d**) The intensity profiles of the probe-1 and probe-2 images versus the control-2 intensity.

Next, we demonstrate that the stored image can be traced simultaneously in its initial and transferred spatial-frequency channels. The pulse sequences of this operation are shown in Fig. 4(a). In this case, two control fields are used simultaneously to read out the stored information. Due to the interaction between two read control fields and stored atomic spin coherence, two probe fields are retrieved. Figure 4(b) shows retrieved images in two information channels for different control-2 intensities. In experiment, the control-1 intensity is kept constant, and the control-2 intensity is changed. It is seen that the transverse profiles of retrieved probe-1 and probe-2 fields both exhibit the three-stripe image. Retrieved probe-1 and probe-2 images come from the same source, i.e. stored atomic spin coherence. The transverse image is transferred into a superposition of the probe-1 and probe-2 modes by using two read control fields in the retrieval process. When two read control fields are used in experiment, the initial single-lambda system is converted into the double-lambda system. In such EIT system, the intensity of each retrieved pulse carrying the image is proportional to that of the corresponding control field^28^. Thus, the image intensity in two information channels can be controlled by changing that of the read control field. Figure 4(c,d) show the intensity profiles of probe-1 and probe-2 images versus the control-2 intensity. It is found that, when the control-2 intensity increases, the intensity of the probe-2 image becomes stronger and that of the probe-1 image becomes weaker. The variation of the image intensity directly mirrors the variation of the pulse intensity of Ref. 28.

## Discussion

We have experimentally demonstrated a controllable spatial-frequency routing of image via stored atomic spin coherence in an EIT-driven crystal. The transverse image carried by the probe pulse is stored into atomic spin coherence by dynamical EIT. By manipulating the spectrum of the read control field, the stored image can be transferred into a new spatial-frequency channel. In addition, the stored image can be converted into a superposition of two spatial-frequency modes by using two read control fields to scatter stored atomic spin coherence. This image routing allows us to manipulate image information in a coherent and all-optical fashion, and will be useful for linking different information channel and devices.

## Methods

The Pr:YSO crystal with dopant concentration of 0.05% is placed inside a cryostat cooled to the temperature of 3.5K. The crystal b axis along 3mm is chosen as the light propagation direction. A single-mode dye laser with 1 MHz linewidth is used as the initial laser source. The laser wavelength is adjusted to the 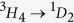 optical transition of 605.977 nm. The initial laser is divided into the required laser beams by beam splitters. Each laser beam passes through acousto-optic modulator (AOM), which allows us to independently manipulate the frequency, intensity and pulse sequence of the corresponding light field. Through lens, all laser beams are made to overlap spatially in the crystal. The angle between laser beams is about 85 mrad. The probe-1 field passes a spatial mask to provide a transverse image. The probe beam passing through the crystal is further divided into two beams: one is guided to a photodiode (PD) monitoring the pulse intensity in the time domain; and the other is guided to a CCD camera monitoring the transverse image in the spatial domain.

## Additional Information

**How to cite this article**: Wang, L. *et al.* Image routing via atomic spin coherence. *Sci. Rep.* **5**, 18179; doi: 10.1038/srep18179 (2015).
